# Investigating the effect of G-Bond and Z-PRIME Plus on the bond strength between prefabricated zirconia posts and the root canal wall in vitro

**DOI:** 10.15171/joddd.2018.020

**Published:** 2018-06-20

**Authors:** Ezzatollah Jalalian, Nahid Darvish, Sogol Saberi

**Affiliations:** ^1^Department of Fixed Prosthodontics, Member of Dental Materials Research Center, Dental Branch of Islamic Azad University, Tehran, Iran; ^2^Dentist, Tehran, Iran; ^3^Dentist, Laser Research Center of Dentistry, Dentistry Research Institute, University of Medical Sciences, Tehran, Iran

**Keywords:** G-Bond, Z-PRIME plus, bond strength, zirconia post, push out test

## Abstract

***Background.*** The aim of this study was to analyze the effect of G-Bond and Z-PRIME Plus on the bond strength between prefabricated zirconia posts and the root canal wall.

***Methods.*** The study was carried out on 21 premolar teeth with similar conditions. The samples were cut at the CEJ. After root canal treatment of the roots, the post space was prepared up to a length of 10mm. The samples were randomly assigned to two groups of 10. G-Bond was used in one group and Z-PRIME Plus in the other to prepare the posts’ surfaces. After cementation and mounting the samples in polyester, the post was cut from the apical area into three equal sections. The bond strength of the samples was tested using the push-out test on a universal testing machine. Data wereanalyzed using t-test.

***Results.*** The meanpush-out bond strengths in the control,G-Bond and Z-PRIME Plus groups were 14.3, 27.6±11.8 and 27.4±13.4N, respectively. There was no statistically significant difference between the two groups (P<0.9). Both methods of surface treatment increased bond strength. The bond strengths were different in different sections such as coronal, middle and apical in each group.

***Conclusion.*** There was no significant differencebetween the G-Bond and Z-PRIME Plus groups and both products in-creased the bond strength of prefabricated zirconia posts.

## Introduction

The bond strength of non-metallic posts to root walls is one of the most important factors in choosing the best technique for repairing endodontically treated teeth that have lost most of their coronal structure. Use of posts to distribute torqueforces along the long axis of the root andprotect against the forces within the oral cavity in restoring endodontically treated teeth has always been a prominent issue.^1^

Teeth that undergo root canal therapy face a higher chance of fracture due to widespread destruction of their structure and these teeth require post space preparation to place a post within the root, which in turn weakens the remaining tooth structure further. In addition, there is the possibility of creating micro-fractures and perforations during this procedure.^2^ In recent years, many types of non-metallic posts have been introduced such as ceramic zirconia posts which serve as an alternative for prefabricated metallic posts. Many advantages have been listed for these posts such as their tensile strength, elastic modulessimilar to that of dentin, conservative canal preparation due to chemical bond to the canal and easier removal process in case of treatment failure and need for retreatment.^3-5^

A study on FRC posts with zirconia coverageshowed that these posts contained 70% glass fiber with parallel orientation, which increased the strength of these posts further and reduced cervical fractures. Another advantage of these posts is the complete polymerization of the cement in deep areas of the canal. The surface of these posts is covered with silane, resulting in a strong bond between the cement and composite. Furthermore, this system includes the smallest posts, which means less preparation is needed in the root canal.^6^

A disadvantage related to zirconia posts is their weak bond to resin-based cements. In order to increase the bond strength, the post needs surface treatments such as lasers, sandblasting and primers.^7^

Recent studies have shown that sandblasting using Cojet and alumina particles increases the bond strength of fiberglass and zirconia posts. Generally speaking, the bond strength of resin-based cements to posts is influenced by the post material and surface treatment.^8^ Also use ofCO_2_ and Nd:YAG lasers, compared to other surface treatment methods such as sandblasting, does not increase the bond strength of zirconia posts to resin-based cements.^9^

According to recent studies, the bond strength between resin-based cements and posts can be controlled by different surface treatments based on the surface hardness and in the future high strength zirconia posts can be used in restorations.^10^

Based on previous studies, zirconia-based primer (Z-PRIME Plus) increases the push-out bond strength between zirconia posts and dentin of the root canal wall.^11^

In addition, use of this primer along with air abrasion as surface treatments increases the shear bond strength between ceramic posts and resin-based cements.^12^

Considering that zirconia posts do not bond well to resin-based cements, surface treatment seems necessary to create a strong bond. The aim of this study was to investigate the effect of G-Bond and Z-PRIME Plus on the bond strength between prefabricated zirconia posts and root canal walls.

## Methods

This was as an experimental study carried out in vitro on 21 mandibular premolar teeth which had specific conditions (caries-free, without fractures or internal resorption and similar age range). The teeth that were used in the study had been extracted for orthodontic reasons and informed consent was obtained from the subjects. After this research, they were not used in any other way and were thrown away. The teeth were without names and could not be assigned to any individual or individuals.

**Figure 1:**
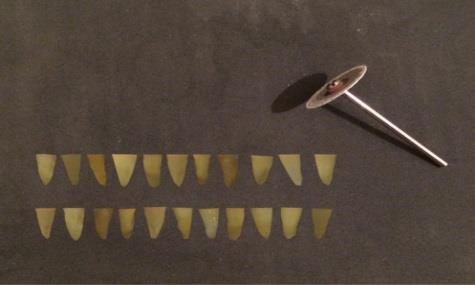
Cutting the crown from CEJ with metal disc.

The teeth were immersed in 0.5% chloramine T solution (Chloramine T trihydrate, Merck KGaA, Darmstadt, Germany) for disinfection and stored in 37°C physiologic serum until used for the purpose of the study. The tooth crowns were cut horizontally at CEJ level using a 0.2-mm-thick metal disc (Prodontholliger, France) and water was used as the coolant (Figure 1).

**Figure 2:**
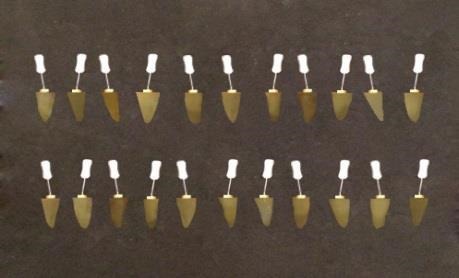
Preparation of canals for post.

The working length of all the root canals was determined using radiography and all the root canals were filed up to #35 K-file (Maillefer) and irrigated frequently with 5.25% sodium hypochlorite solution. The MAF for all the teeth was considered file #45. Then the teeth were flared up to file #60 using the step-back technique (all the files were from the Maillefer Company and were 25mm in length)(Figure 2).

**Figure 3:**
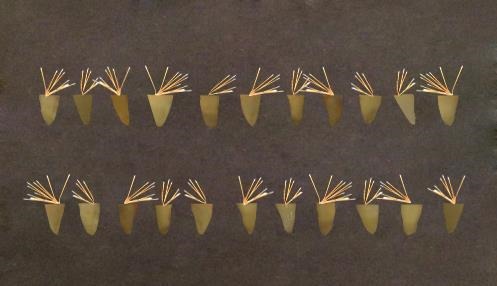
Obturation of canals.

The canals were dried with paper cones. The master cone for all the root canals was chosen at cone #35 and the lateral cones were #15 cones. Obturation was performed using the lateral condensation technique with a #25 finger spreader (Maillefer) and AH-26 sealer (DentsplyDeTrey GmbH-Germany) such that the finger spreader was packed at 1mm shorter than the working length (Figure 3).

After obturation, the teeth were divided randomly into two groups of ten and 1 tooth was considered as the control sample.^1,3,13,14-22^ The posts and materials used in each tooth arepresented in Table 1.

**Table 1:** The dental material used and their manufacturer

**Dental Material**	**Manufacture**
Ice Light (FRC post coated by zirconia)	Danville
Adhesive system (Dual cure) Panavia F_2_	Kuraray, Japan
Sealer AH-26	DentsplyMaillefer
G-Bond	GC JAPAN
Z-PRIME Plus	BISCO USA

**Figure 4:**
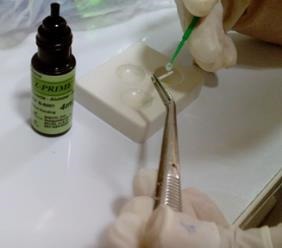
Surface treatment of posts with Z-PRIME bonding agent.

In one group the zirconia post was treated with Z-PRIME Plus (Figure 4). The post space was prepared using #1, #2 and #3 piezo burs respectively and then a universal drill was used. The post space was 10mm in length and 3‒5mm away from the apex.^14,15^ Then the finishing drill #3 was used to perform final preparations. A rubber stop on the drill handle was used as a guide in preparing the canal to a specific length based on radiographic findings. The remaining material within the canal was then removedusing a water spray and an air syringe without water and oil. Then the posts were tested withinthe canal to ensure a passive fit.^23-25^ Using a 008 diamond fissure bur with water cooling, the tip of the posts was cut off. The post space was dried using paper cones and etched with37% phosphoric acid for 15 seconds. Then the canal was rinsed and dried without complete dehydrationand had visible moisture (dried for 3 seconds).^6^

Z-PRIME Plus was prepared according to manufacturer’s instructions and applied to the zirconia post’s surface using a micro-brush. Then the cement liquids A and B were mixed according to manufacturer’s instructions and applied withinthe canal wall with a micro-brush and the mixture was dried gently with an air syringe for 30 seconds. Excess material was removed using paper cones. Then appropriate amounts of pastes A and B were mixed and the post’s surface, already prepared with Z-PRIME Plus, was completely covered with cement. The post was then placed within the root canal. The excess cement was again removed and the cement was light-cured for 60 seconds and in order to ensure complete setting of the cement, Oxyguard was placed on the canal orifice for 3minutes.^27^

**Figure 5:**
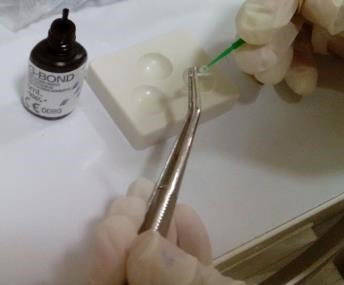
Surface treatments of posts with G-bond.

In the other group, all the steps stated above were repeated except that Z-PRIME Plus was replaced by G-Bond to prepare the surface of the zirconia post (Figure 5). The control tooth did not receive any surface treatment.

**Figure 6:**
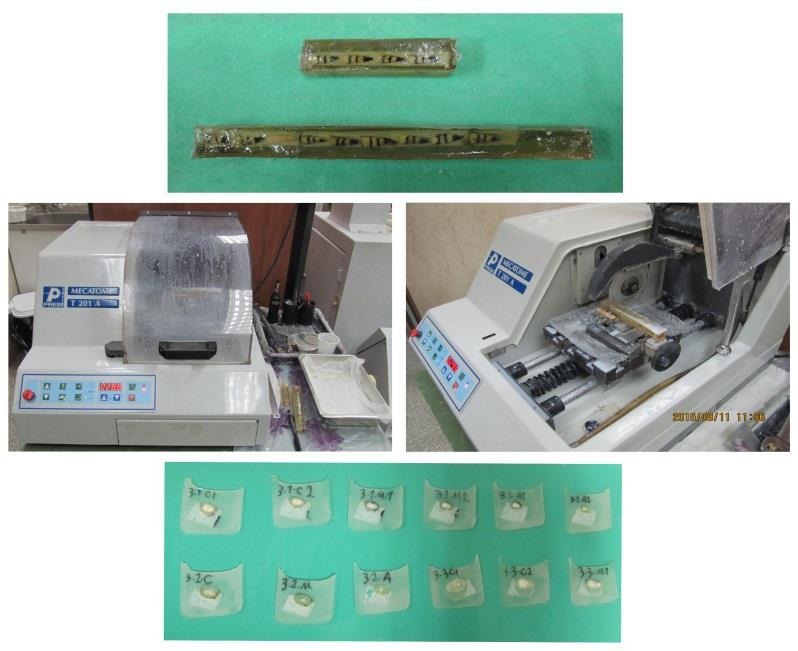
Cutting and mounting of specimens.

Twenty-four hours after cementation and mounting of the samples in polyester, the post was cut into three equal apical, middle and coronal pieces from the apical area using a cutting machine (Figure 6).^28^

**Figure 7:**
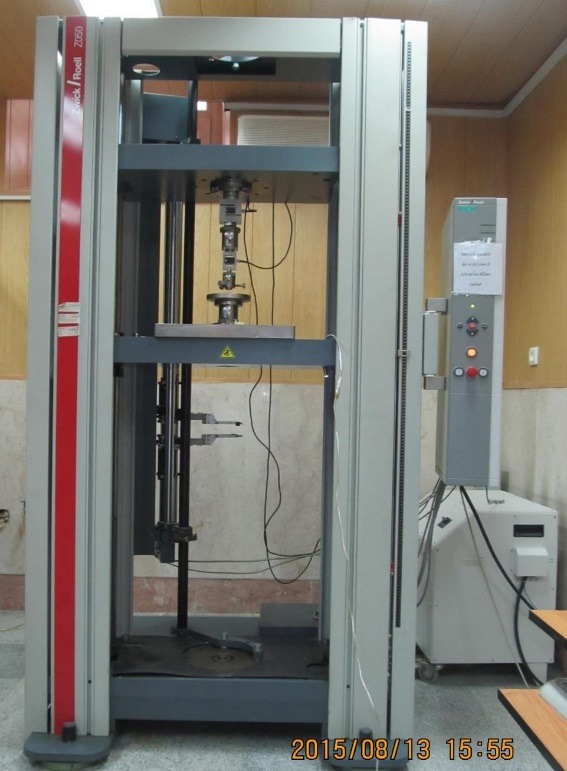
Push out test.

After sample preparation was complete, the bond strength for each section was tested using the push-out method with the Instron testing machine at a crosshead speed of 0.5mm/min (Figure 7).

The testing rod had a tip measuring 0.8mm in diameter; the force was applied in an apical-to-coronal direction. The point, at which the curve which was being drawn by the computer recording the data dropped, was considered the debonding point. The experiment was carried out in the laboratory of Tehran University Research Center. Because of the normal distribution of data, the t-test was used for analysis.^16,17^

## Results

This study was carried out on 21 premolars, 10 prepared with G-Bond and 10 with Z-PRIME Plus and one control tooth with no surface treatment.

The bond strength of the control sample was 14.3 N.

The bond strengths of samples are shown in Table 2 in terms of the type of surface treatment used.

**Table 2:** Bond strength based on the type of preparation

**Type of preparation**	**Bond strength**	
	**Amount**	**CV**
**G-Bond**	27.6 ± 11.8	43
**Z-PRIME Plus**	27.4 ± 13.4	46
**Test result**	P < 0.9	

The mean bond strength values of the G-Bond and Z-PRIME Plus groups were 27.6 and 27.4 N, with no statistically significant difference (P<0.9).

The standard deviations in the G-bond and Z-PRIME Plus groups were 43 and 46, with no significant difference.

The bond strengths in the G-bond and Z-PRIME Plus groups were almost twice that of the control sample. This proves that surface treatment was effective.

The bond strength in each section (apical, middle, coronal) was measured in terms of the type of preparation.

Bond strength of the G-bond group in the coronal, middle and apical sections were 34, 30.1 and 25.5 N, respectively; in the Z-PRIME Plus group these values were 35.5, 31.7 and 20.7 N, respectively. The bond strength in both groups was similar in these three sections. In both groups the bond strength decreased as from the coronal section to the apical section. The bond strength droped from 34 to 25 N in the G-bond group and from 35.5 to 20.7 in the Z-PRIME Plus group.

**Figure 8:**
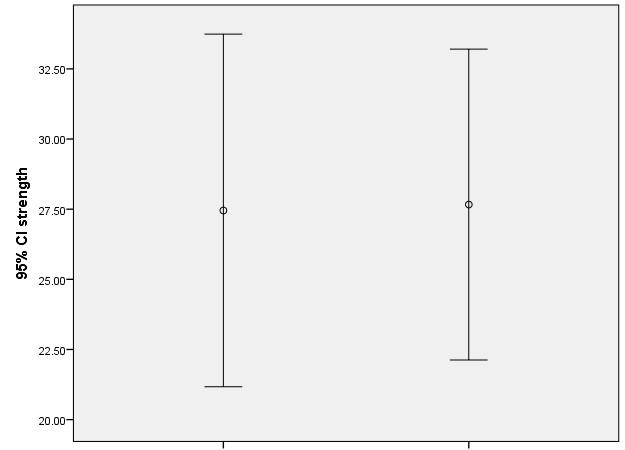
Bond strength diagram for G-Bond and Z-PRIME Plus preparation.

Figure 8 shows the amount of force needed to debond the samples.

## Discussion

This study showed no statistically significant differences between the bond strength of the two groups with different preparation materials, G-Bond and Z-PRIME Plus. It was also reported that both groups exhibited approximately twice the bond strength of the control sample; thus when preparing zirconia posts, if the post is treated with either material a significant increase in bond strength and retention is achieved and the stability of endodontically treated tooth is increased.

In a study by Korkmazet al^18^ in 2014, the effect of surface treatments such as sandblasting, grinding, primers and alloying on the bond strength between zirconia and ceramic crowns was analyzed. Similar to the results of the present study, it was reported that use of chemical agents to improve bond strength proved to be more effective than mechanical methods.

Torabi et al^11^ conducted a study in 2015 on the effect of a new zirconia primer (a mixture of organophosphate and carboxylate monomers) on the push-out bond strength between zirconia ceramic posts and dentin of the canal wall. Two resin-based cements were used in the study, Panavia F and Clearfil SA with and without using the zirconia primer (Z-PRIMER Plus; Bisco). The push-out test was used to test the bond strength. Based on the results, the zirconia primer (Z-PRIMER Plus) significantly increased the bond strength between zirconia posts and dentin of the canal wall, consistent with the results of the present study.

In a study by Ahn et al^12^ in 2015, the effect of phosphate monomer-containing primers on the shear bond strength of Y-Tzp ceramics and MDP-containing resin-based cements was investigated. Similar to this study, Z-Plus was used to prepare the surface of zirconia. The results showed that use of MDP-containing primers (Z-PRIMER Plus) is an efficient method to increase the shear bond strength of Y-Tzp zirconia and resin-based cements, consistent with the results of the present study.^12^

In another study by Fischer et al^21^ in 2008, the effect of sandblasting and silica coverage of zirconia surfaces on the bond strength of ceramic crowns to zirconia was investigated. In this study the surface morphology of zirconia was examined after various methods of surface treatment using scanning electron microscopy. Similar to the current study, a universal testing machine was used to perform the push-out test at a crosshead speed of 1mm/min to test the bond strength of the samples. The results showed that the bond between ceramic veneers and zirconia were more chemical-based than mechanical. Also surface treatment of zirconia increased the bond strength between zirconia and ceramic crowns, confirming the results of the present study.^21^

Pahlevan et al^20^ conducted a 2011, in whichthe shear bond strength between zirconia posts and canal wall dentin was investigated with two different cements, and zirconia and fiber posts were compared. The cements used were zinc phosphate cement and Panavia F2 cement. In the present study, too, the cement used was Panavia F2. The push-out test was used to analyze bond strength. Based on the results, the bond strengths of Panavia cement and zirconia post were higher than the bond strength of zinc phosphate cement and fiber post, consistent with the results of the present study. They reported that use of non-prefabricated (custom) zirconia posts and resin-based cements can result in a higher bond strength compared to fiber posts; therefore, the passive fit resulting from the matching of the post and post space is an important factor in retention, and bonding alone should not be relied on to achieve retention. This report also verifies the results of the present study. The difference between the present study and the study above was the use of custom or non-prefabricated zirconia posts which were replaced with prefabricated zirconia posts in our study; however, even with this difference, the similarities between these two studies can, to some extent, justify the similarities between the results.

Based on the results of a study by Li et al^8^ in 2015, and also the results of a study by Mirzayi et al^9^ in 2008, sandblasting as a method of surface treatment for posts can increase the bond strength between zirconia posts and resin-based cements because of surface roughness left in place after this procedure. Generally speaking, the bond strength depends on the material of the post and the surface treatment used.^10^ The results of this study emphasize the use of surface treatment on zirconia posts in order to increase the bond strength and in this sense they verify the results of the present study.

According to the results of a study by Gargariet al^19^ in 2011, which was a review of all theinternational papers that examined cementation of zirconia restorations in clinical applications, surface treatment was reported to be one of most efficient methods for increasing bond strength between resin-based cements and zirconia restorations, consistent with the results of the present study.

Another important finding of this study is that the bond strength in both G-bond and Z-PRIMER Plus groups decreased from the coronal to the apical section.

## Conclusion

According to the results of the present study, there was no statistically significant difference between the bond strength of the G-Bond and Z-PRIMER Plus groups. Both these groups increased the bond strength; therefore, it can be clinically beneficial to use them as a surface treatment agent for zirconia posts. In addition, this improvement in bond strength was higher in coronal sections.

## Authors’ contributions

EZ was responsible for the study design. ND was responsible for drafting and running the project. SB was responsible for revising and running the project.

## Acknowledgments

Not applicable.

## Funding

This study has no funding source (Not applicable).

## Competing interests

The authors declare no competing interests with regards to the authorship and/or publication of this article.

## Ethics approval

Not applicable. This study was in vitro. The teeth that were used in this study were extracted for orthodontic reasons and informed consent was obtained from the patients. After this research, they were not used in any other way and were thrown away. The teeth were without any names and could not be traced back to any individual or individuals.
